# A New Active Substance Derived from Lyzed *Willaertia magna* C2c Maky Cells to Fight Grapevine Downy Mildew

**DOI:** 10.3390/plants9081013

**Published:** 2020-08-11

**Authors:** Sandrine Demanèche, Laurène Mirabel, Olivier Abbe, Jean-Baptiste Eberst, Jean-Luc Souche

**Affiliations:** R&D Department, Amoéba, 69680 Chassieu, France; laurene.mirabel@amoeba-biocide.com (L.M.); olivier.abbe@amoeba-biocide.com (O.A.); jean-baptiste.eberst@amoeba-biocide.com (J.-B.E.); jean-luc.souche@amoeba-biocide.com (J.-L.S.)

**Keywords:** *Plasmopara viticola*, biofungicide, *Willaertia magna* C2c Maky, downy mildew, grapevine

## Abstract

Downy mildew of grapevine is one of the most destructive grapevine diseases worldwide. Nowadays, downy mildew control relies almost exclusively on the use of chemical pesticides, including copper products, which are efficient but controversial due to their environmental toxicity. Natural plant protection products have become important solutions in the quest for the sustainable production of food and pest management. However, most biocontrol agents currently on the market, such as biofungicides or elicitors, have a limited efficacy; thus, they cannot replace chemical compounds in full. Our innovation is a natural active substance, which is a lysate of the amoeba *Willaertia magna* C2c Maky. This active substance is not only able to elicit grapevine defenses, but it also demonstrates direct fungicidal activity against *Plasmopara viticola*. The efficacy of this new natural substance was demonstrated both in a greenhouse and in a field. The amoeba lysate provided up to 77% protection to grapevine bunches in the field in a natural and safe way.

## 1. Introduction

The grapevine industry, which represented 7.4 million hectares of crops worldwide in 2018 and is mainly spread over five countries (Spain, China, France, Italy, and Turkey) [1], is highly affected by downy mildew of grapevine, one of the most destructive grapevine diseases worldwide [2]. Indeed, it can decrease the crop yield by over 30%, and can also have an impact on fruit and wine quality [3]. The downy mildew of grapevine, probably introduced into Europe in the 1870s from infected American grafts used to replant the French vineyards destroyed by Phylloxera, is caused by *Plasmopara viticola* [4]. Described for the first time in 1834 from north-eastern United States vine samples and finally identified as *P. viticola* in 1888 [4], this micoorganism is an obligate biotrophic oomycete that affects every green and young grapevine organ [5]. During the winter season, the pathogen *P. viticola* is mainly preserved in oospore shape in soils, waiting for spring. When the temperatures reach more than 11 °C and when the rain falls, oospores germinate and zoospores are produced, which contaminate young grapevine organs [6]. 

Currently, the vine cultivars mainly used in Europe are not resistant to this disease. Although some resistant varieties exist, they have not been able to find a place in the market until now [7]. Currently, downy mildew control relies almost exclusively on the use of chemical pesticides, including copper products, which are efficient but controversial due to their environmental and human toxicity [4,7]. Although some curative fungicides exist, resistance toward these quickly emerged. Current strategies to control grapevine downy mildew are based on the use of preventive fungicide treatments, among these, copper and mancozeb are still widely used. Control of grapevine downy mildew can also be achieved by mixing or alternating products containing active substances with different mode of actions to increase disease control and reduce the risk of resistance from the beginning of the period during which plants are susceptible to infection [4]. A total number of 10 annual applications, or even more on rainy years, is a very common practice in some regions, such as the southwest of France (Bordeaux). Some studies have shown that copper in soil accumulates after several applications, increasing copper availability, which can become excessive and cause grapevine damage, such as physiological changes and reduced photosynthesis rates and plant growth [8].

Current European environmental plans require reducing chemical pesticides in crop treatments and replacing them with natural substances [9]. Moreover, by the end of 2019, the European Commission renewed the authorization of copper for only seven years and limited its use to an average of 4 kg per hectare per year. It is possible that it can be withdrawn after 2026, as is already the case in Denmark and the Netherlands. Moreover, Mancozeb, a key standard fungicide on both grapes and potatoes, could also be banned as early as 2021. According to this European trend, and due to the need for other solutions for organic crops, biological alternatives are in high demand.

In this context, Amoéba, a young and innovative company, has developed a natural product based on the amoeba *Willaertia magna* C2c Maky to control downy mildew of grapevine. *W. magna* C2c Maky, a thermophilic free-living amoebae (FLA) belonging at the Vahlkampfiidae family [10], was isolated from the water of a thermal swimming pool in 1988. The lack of pathogenicity of this amoeba was demonstrated by culture and confirmed by omic analyses [11,12]. FLA are ubiquitous protozoa that inhabit common aquatic environments [13,14]. FLA are predatory and consume bacteria for their growth [15,16,17]. All published papers on FLA deal with the living form of the amoebas and their phagocytic activities. For the first time, we report a potential application of the dead form of *W. magna* C2c Maky (lysate) in the domain of plant protection products. The product developed by Amoéba is derived from the dead form of amoeba, lysed by high-pressure homogenization. This is the first report to our knowledge of the development of a fungicide product based on a free-living amoeba. This paper highlights the innocuity and the efficacy of the lysate of *W. magna* C2c Maky against the downy mildew of grapevine, both in a greenhouse and in a field, and presents its dual mode of action. 

## 2. Results

### 2.1. Elicitor Property

#### 2.1.1. Comparative Analysis of Grapevine Gene Defense Induction

To determine, in controlled conditions, whether *W. magna* C2c Maky lysate has properties to induce plant defense genes in grapevine plants, the qPFD^®^ system based on 28 marker genes was used [18]. The genes were selected as being representative of the main defense pathways in plants, from the genes involved in parietal modification to those in the signaling pathways (i.e., salicylic acid, jasmonic acid, and ethylene).

**Figure 1:**
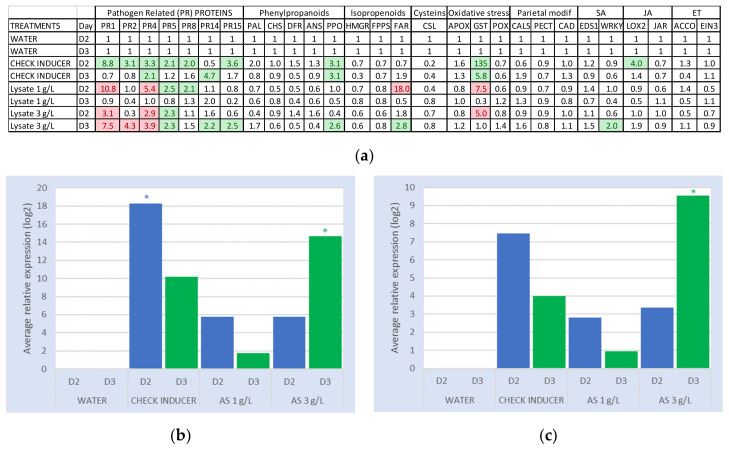
Elicitor effect of the active substance (AS): (**a**) Fold change represents relative gene expression values (2−ΔΔCt) of the defense genes (qPFD^®^) in grapevine, compared to the water control. Values superior or equal to 2 are in green (moderate inductions), and the ten highest values of the lysate data are in red (high inductions). Assessment of the candidate product at two doses, compared to the internal check inducer, at two (D2) and three (D3) days after treatment (D0). A H_2_O_2_ application was performed 24 h later (D1) in order to mimic a pathogen attack. The average relative expressions were obtained by RT-qPCR from two independent repetitions and relative to the water control at each sampling date. The results are the average of the relative expressions of genes from two independent repetitions. SA, salicylic acid; JA, jasmonic acid; ET, ethylene. Complete gene names are provided in Table 1. (**b**) The cumulation of inductions (log2 relative expressions > 0) of the 28 defense genes in the grapevine qPFD^®^, for amoeba lysate compared to the internal check inducer, at the sampling dates D2 and D3. The sum of inductions represents that the sum of the average relative expressions (log2 (2−ΔΔCt)) is higher than zero for the 28 genes, relative to the water control, at each sampling date. (**c**) Cumulation of inductions (log2 relative expressions > 0) of the pathogen-related (PR) protein genes from the grapevine qPFD^®^, for amoeba lysate compared to the internal check inducer, at the sampling dates D2 and D3. The sum of the inductions represents the sum of the average relative expressions (log2 (2−ΔΔCt)) of the PR protein genes with values higher than zero, relative to the water control, at each sampling date. *: *p* < 0.05.

In this experiment, the internal check inducers ChitoOlygoSaccharides and OligoGAlacturonide (COS-OGA) [19] showed a low to moderate induction capacity of pathogen-related (PR) protein genes. It also induced the glutathione S-transferase (GST) gene at a high level, especially at the sampling date of two days after treatment (D2) (Figure 1a). Overall, the gene induction levels were higher at D2 than at D3 (three days after treatment) for the check inducer. The amoeba lysate, at a dose of 3 g/L, induced mainly PR protein genes at the sampling date D3, whereas at a dose of 1 g/L, it induced some PR protein genes, mainly at the sampling date D2 (Figure 1a). The polyphenol oxidase (PPO) gene was moderately induced at a dose of 3 g/L and not induced at 1 g/L, whereas the (E,E)-alpha-farnesene synthase (*far*) gene was highly induced at D2 by the lysate at 1 g/L and moderately induced at D3 by the lysate at 3 g/L.

Analysis of the cumulation of inductions of the 28 genes confirmed that the internal check inducer had a moderate induction effect, higher at the sampling date D2 than at D3, with values around 18 and 10, respectively (Figure 1b). The amoeba lysate, at a dose of 3 g/L, had a low induction effect at D2 (<10), lower than the internal check inducer. At D3, it showed a moderate induction effect (>10), slightly higher than the internal check inducer at the same date. At a dose of 1 g/L, it had globally a low induction effect at D2 (<10) and did not show any significant induction capacity at D3.

In the same way, analysis of the cumulation of inductions of the PR genes showed that the internal check inducer demonstrated a low to moderate induction capacity of these genes, higher at the sampling date D2 than at D3, with values around 7 and 4, respectively (Figure 1c). The amoeba lysate, at a dose of 3 g/L, had a low induction effect at D2 (<5), lower than the internal check inducer and not significantly different from the water control. At D3, it showed a significant induction effect (*p* < 0.05), higher than the internal check inducer on the same date. At a dose of 1 g/L, it had a low induction effect at D2 (<5) not significantly different from the water control (*p* > 0.05), and did not show any significant induction capacity at D3.

#### 2.1.2. Fold Change of PR Protein Genes

The analysis will now focus on the fold change values of the PR protein genes at the sampling dates D2 and D3. Fold change represents the relative gene expression values (2−ΔΔCt) without log2 transformation (Figure 1a), compared to the water control and averaged for both repetitions. The internal check inducer showed a moderate induction of most of the PR protein genes, 2 to 9 times higher than the water control, at D2. At D3, its induction capacity decreased: only the PR4 and PR14 genes were induced, 2 and 5 times higher than the water control, respectively (Figure 1a). Amoeba lysate, at a dose of 3 g/L, showed a better induction capacity of the PR protein genes at D3 compared to D2, with induction levels 2.2 to 7.5 times significantly higher than the water control (*p* = 0.024). At a dose of 1 g/L, it did not show any significant induction effect on the PR protein genes at D3, except for the PR14 gene, which was 2 times more induced than the water control. At D2, it induced the PR8, PR5, PR4, and PR1 genes, 2 to 11 times higher than the water control (Figure 1a).

### 2.2. Anti-Oomycete Activity

**Figure 2:**
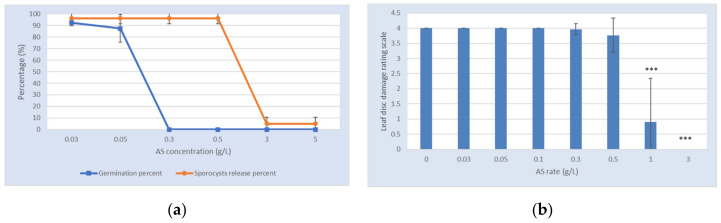
The direct effect of the active substance (AS). The results are expressed as the mean +/− standard deviation. (**a**) Evaluation of sporocyst release and zoospore germination in microplates in the presence of increasing doses of AS on *P. viticola*. The orange line represents the percentage of sporocyst release, while the blue represents the percentage of zoospore germination. The AS concentration ranged from 0.03 to 5 g/L. (**b**) Evaluation of the damages caused by *P. viticola* in the presence of increasing doses of AS on leaf grapevine discs. The AS concentration ranged from 0.03 to 3 g/L. The leaf disc damage rating scale is an arbitrary scale ranging from 0 (no damage) to 4 (completely damaged). ***: *p* < 0.001.

The aim of this study was to screen the efficacy of the active substance (AS) against grape downy mildew (*P. viticola*) under in vitro and in vivo conditions. For the in vitro study, two criteria were studied, namely, sporocyst release and zoospore germination. A dose response was obtained for the sporocyst release; the percentage of the release ranged from 96% to 5% for the studied concentrations from 0.03 to 5 g/L (Figure 2a). For zoospore germination, the percentages ranged from 92.5% to 0% for concentrations from 0.03 to 5 g/L, respectively. No germination was observed from 0.3 g/L (Figure 2a).

For the in vivo study, three criteria were evaluated, namely, the sporulation of the pathogen mixed with the product, the total number of sporocysts produced, and the contaminating capacity of the sporocysts produced after treatment with the AS. The AS at 1 and 3 g/L showed significant control (*p* < 0.001) of the sporulation on leaf discs (Figure 2b), with 77.5% and 100% inhibition, respectively. Concerning the production of sporocysts of *P. viticola* mixed with the AS, for all of the studied rates, except at 3 g/L, sporocysts were produced, ranging from 246,000 sporocysts at 1 g/L to 1,230,000 sporocysts at 0.03 g/L. The sporocyst production decreased when the quantity of the AS increased. However, whatever the concentration of product applied, the produced sporocysts were able to provide further contamination, except at 3 g/L, where no sporocysts were produced.

### 2.3. Greenhouse Tests

**Figure 3:**
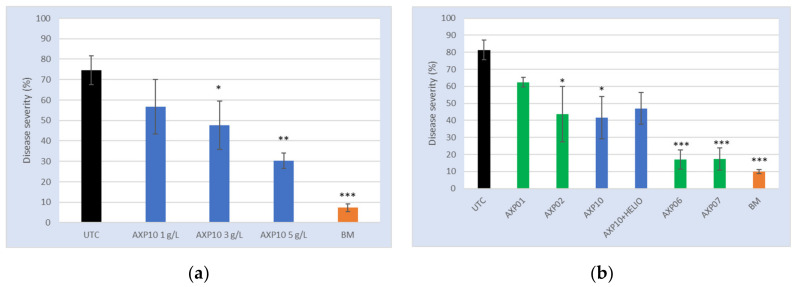
Greenhouse tests: (**a**) Disease severity evaluated on leaves L4 to L2 treated with AXP10 at 1, 3, and 5 g/L, and the Bordeaux mixture (BM) as the reference product. Leaves are numbered from the top of the plant (L1 being the youngest at the top). The untreated control (UTC) was spread with demineralized water. (**b**) Comparison of the formulated products AXP01, AXP02, AXP06, AXP07, AXP10 + HELIOSOL^®^, and pure active substance (AS) AXP10 at a rate of 3 g/L of AS. AXP01 and AXP02 contained 50% AS and were prepared at 6 g/L, while AXP06 et AX07 contained 62.5% AS and were prepared at 4.8 g/L. HELIOSOL^®^ was used at a rate of 1.25 mL/L, while BM was used at 6.25 g/L. *: *p* < 0.05; **: *p* < 0.01; ***: *p* < 0.001.

The goal was to evaluate the sensitivity of *P. viticola* to increasing the doses of a product containing 100% AS (product code AXP10) under greenhouse conditions. Treatments were applied in a preventive way, 24 h before contamination with a *P. viticola* strain sensitive to the main fungicide families used to control grape downy mildew. The level of the disease severity in the untreated control (UTC) was 74.7%, meaning that 74.4% of the leaf area was affected by disease. A 7.3% disease severity was observed in the plant treated with the Bordeaux mixture (BM). The disease severity for AXP10-treated plants decreased from 56.7% to 30.3% for concentrations 1 and 5 g/L, respectively (Figure 3a). Thus, a clear dose response was observed with a significant protection for the two highest doses (*p* = 0.043 and *p* = 0.0031, respectively).

The efficacy of the product AXP10 (containing 100% AS) was compared to the formulated products AXP01, AXP02, AXP06, AXP07, and AXP10 + HELIOSOL^®^ (ACTIONPIN, Castets, France). HELIOSOL^®^ is an eco-adjuvant for plant protection treatments, formulated with pine derivatives; it improves the effectiveness of fungicides, herbicides, insecticides, and growth regulators on all crops. The formulated products were wettable powders. AXP01 and AXP02 contained 50% AS, and AXP06 and AX07 contained 62.5% AS. An 81.3% disease severity was observed in the untreated control, whereas a 10% disease severity was observed with 3.75 kg/ha of BM. AXP06 at 4.8 g/L and AXP07 at 4.8 g/L (3 g/L of AS) showed a disease severity almost comparable to 3.75 kg/ha of BM providing a significant protection to the grapevine (*p* < 0.001). AXP02 at 6 g/L (3 g/L of AS), AXP10 at 3 g/L, and AXP10 at 3 g/L with 1.25 mL/L of HELIOSOL^®^ were comparable (disease severity between 41.7% and 47%), but only AXP02 and AXP10 alone were proven to be statistically different from the untreated control (*p* < 0.05). AXP01 at 6 g/L (3 g/L of AS) was by far the least effective treatment, with a disease severity close to 62.3% (Figure 3b), not significantly different from the untreated control.

### 2.4. Efficacy in the Field

**Figure 4:**
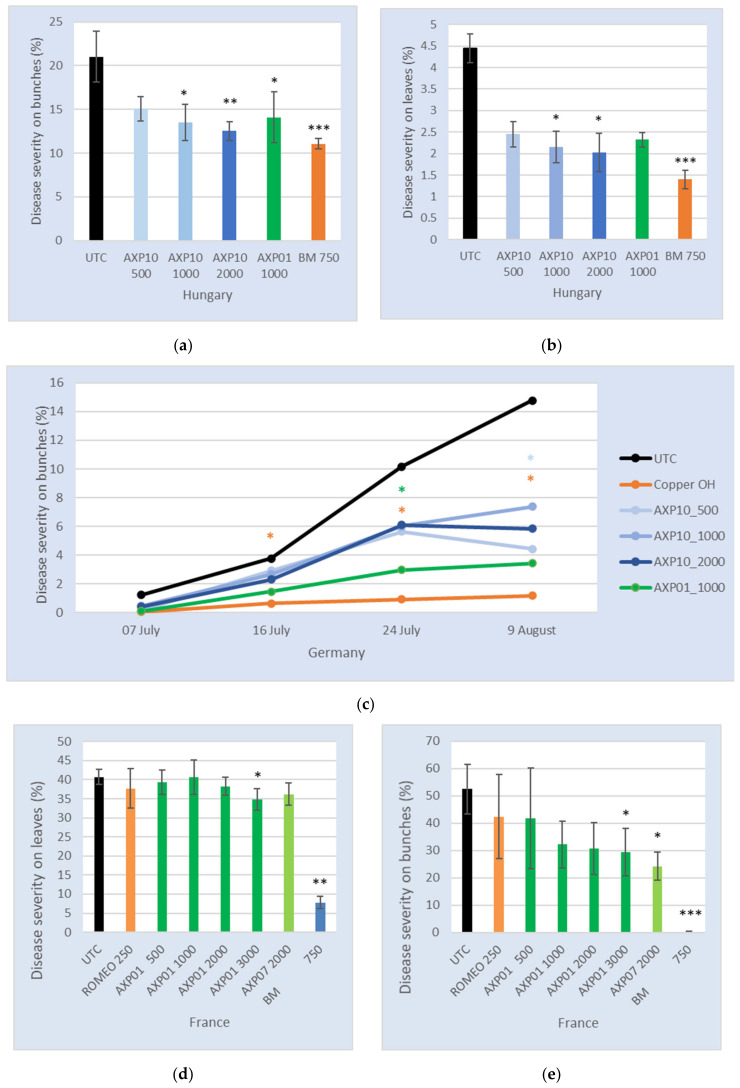
Field tests: Disease severity evaluated on the leaves (**a**) and the bunches (**b**) in a field trial conducted in Hungary. The black represents the untreated control; the increasing color intensities of blue correspond to increasing AXP10 concentrations from 500 to 2000 g AS/ha; the green represents AXP01 at 1000 g AS/ha; and the orange represents the Bordeaux mixture (BM) at 750 g copper/ha. (**c**) Disease severity evaluated on the bunches in a field trial conducted in Germany. The black represents the untreated control; the increasing color intensities of blue correspond to increasing AXP10 concentrations from 500 to 2000 g AS/ha; the green represents AXP01 at 1000 g AS/ha; and the orange represents copper hydroxide at 610 g copper/ha. Disease severity evaluated on the leaves (**d**) and the bunches (**e**) in a field trial conducted in France. The black represents the untreated control; the darker green represents the treatment with AXP01 at concentrations ranging from 500 to 3000 g AS/ha; the lighter green represents AXP07 at 2000 g AS/ha; and the orange represents Roméo^®^ at 250 g/ha and the blue represents BM at 750 g/ha. *: *p* < 0.05; **: *p* < 0.01; ***: *p* < 0.001.

The first trial was set to confirm the dose response observed in a greenhouse by measuring the reduction in the average disease severity in the treated plants compared to the untreated condition. This was conducted in a field trial performed in 2019 in Hungary (near Gyékényes in the southwest of Hungary). The weather conditions were slightly wet, with one rainy episode from 16th June to 24th June, with a peak at 22 mm of rain on 22nd June. The last rating was performed on 4th July and revealed a mildew attack of 4.5% on the untreated leaves and 21% on the untreated bunches. The disease was reduced to 2.5%, 2.2%, and 2.0% on the leaves and to 15.1%, 13.5%, and 12.5% on the bunches in the presence of AXP10 at the rate of 500, 1000, and 2000 g/ha, respectively (Figure 4a,b). A significant protection on the leaves was obtained for the two highest doses of AXP10 (*p* < 0.05, Figure 4b) and on the bunches for all the conditions except for the smallest dose of AXP10 (Figure 4a). The level of protection with the highest dose of AXP10 on the bunches was similar to BM RSR Disperss^®^ (UPL Europe Ltd., Warrington, U.K.) containing 200 g/kg of copper at 750 g/ha (11.1%).

The second trial was set to compare the efficacy of AXP10 at a rate of 0.5, 1, and 2 kg/ha, in parallel with AXP01 at 2 kg/ha, equivalent to 1000 g AS/ha, and with copper oxychloride (Cuprozin^®^ Progress, BIOFA, Germany) against *P. viticola* in a vineyard under natural conditions. This trial was conducted in Germany (located in Sulzfeld in Bavaria) in 2019. Regular episodes of rain were observed throughout the trial, with a probable contaminating event on 20th and 21st May with 16.1 and 11.6 mm rain, respectively, and again on 11th June with 12.8 mm of rain. At the end of the experiment, the disease severity under untreated conditions was 19.5% on the leaves and 14.8% the on bunches. AXP10 was efficacious at the three tested doses (500, 1000, and 2000 g AS/ha) throughout the experiment (from 8th July to 9th August), in decreasing the severity on the leaves and bunches to below 8%, but due to the biological variability, a statistical difference could be measured only for AXP01 at 1 kg/ha on the 24 July and for AXP10 at 0.5 kg/ha on the 9 August (Figure 4c). The formulated product AXP01 demonstrated a better efficacy, maintaining the severity on the leaves and bunches at below 4%.

The third trial aimed to determine the selectivity and the efficacy of AXP01 at a rate of 1, 2, 4, and 6 kg/ha and AXP07 at 3.2 kg/ha against *P. viticola* on grapevine. The references were BM RSR Disperss^®^ (UPL Europe Ltd., U.K.) at 3.75 kg/ha (750 g copper/ha) and Roméo^®^ (Agrauxine, Marcq en Barouel, France) at 0.25 kg/ha. Roméo^®^ active substance is composed of 941 g/kg of cerevisane (yeast wall extracts of *Saccharomyces cerevisiae*). This trial was conducted in a vineyard with an artificial infection in Moulon, in the southwest of France. The cold weather in May was not suitable for disease development; however, at the beginning of June and mid-June, heavy contaminating rain occurred, and the disease began to spread in the trial. A new heavy rain was recorded at the beginning of July. The leaves and the bunches were strongly infested in the untreated plots by the end of the trial (41% severity on the leaves and 52% on the bunches). BM at 3.75 kg/ha brought about significant leaf and bunch protection, with an efficacy of 80.7% and 99.6%, respectively. Roméo^®^ at 0.25 kg/ha showed no significant reduction in infestation on the leaves (9% efficacy, *p* > 0.05) and on the bunches (22% efficacy, *p* > 0.05). AXP01 at 1, 2, 4, and 6 kg/ha and AXP07 at 3.2 kg/ha provided comparable results to Roméo^®^ at 0.25 kg/ha on the leaves (Figure 4d), but the highest dose was significantly different from the control (*p* < 0.05). A better efficacy was obtained on the bunches with a significant protection for the highest dose of AXP01 and for AXP07 (*p* < 0.05, Figure 4e). A dose effect was observed on the bunches for AXP01, with an increased efficacy from 28% at 500 g AS/ha to 42% at 3000 g AS/ha (Figure 4e). AXP07 at 3.2 kg/ha provided the best protection on the bunches, with 52% efficacy (Figure 4e).

## 3. Discussion

The aim of this study was to evaluate the ability of a natural product to fight grapevine downy mildew. The active substance (AS) of this product is a lysate of the amoeba *W. magna* C2c Maky manufactured in Amoéba (Chassieu, France). It can stimulate plant natural defenses and also inhibit *P. viticola* spore germination leading to the protection of the leaves and the bunches of grapevines. The use of *W. magna* C2c Maky as a fungistatic and fungicide agent is subject to patent protection [20]. Based on the toxicity (Table S1) and ecotoxicity (Table S2) study results, “Lysate of *Willaertia magna* C2c Maky” is not classified according to European Regulation (EC) No. 1272/2008 on the classification, labeling, and packaging of substances and mixtures, meaning that the product is harmless to both human health and the environment. Actually, despite the toxicity of copper [21] and mancozeb [22], these two compounds are the most commonly used products to protect grapevine against pathogen attacks [4]. Mathematical models have predicted an increase in disease pressure in the future that would lead to an increase in the number of treatment [23]. Meanwhile, there is an increasing demand for the use of products that are harmless for human health and the environment. Biocontrol products are agents and products that use natural mechanisms to fight bio aggressors. The fungicidal biocontrol agents are based on natural substances such as essential oils or microorganisms. A lot of natural compounds and microorganisms are known to have antifungal properties. Among 103 experimental treatments tested under controlled conditions, 32 were further evaluated in field trials in comparison to a copper reference treatment, but none of these substances were able to fully replace copper [7], reinforcing the need for innovation such as our product. Some microorganisms are known to be efficient at fighting grape downy mildew. It can be in their living form, such as *Fusarium proliferatum* [24,25], *Lysobacter capsici* AZ78 [26], *Bacillus subtilis,* and *B. pumilus* [27], or *Trichoderma harzianum* T39 [28], for example. Field trials conducted from 1992 to 1995 demonstrated a reduction of disease severity, ranging from 53% to 99% depending on the strength of *P. viticola* attack, by year and on the vine cultivar [24]. *Bacillus subtilis* GLB191 is also an efficient biological control agent against *P. viticola*, whose activity results from both a direct effect against the pathogen and the stimulation of plant defenses [29]. The authors managed to identify that the active molecules were surfactin and fengycin, both being lipopeptides. In the same way, *Bacillus subtilis* KS1 produced iturin A, a lipopeptide active against *P. viticola* [30]. *W. magna* C2c Maky is known to possess specific lipopeptides [31] that might be involved in the activity of AS. Further studies are needed to identify the origin of the activity in the lysate of *W. magna* C2c Maky. To our knowledge, this is the only published use of lysed microbial cells in plant protection domain. Cerevisane contained only the purified cellular walls of *Saccharomyces cerevisiae* strain LAS117. The *Bacillus* strains (*amyloliquefaciens, firmus, pumilus* and *thuringiensis*) and other microorganisms are sold as living microorganisms. Working with dead cells is a great advantage in term of storage condition and handling facilities because there is no need to maintain viability. 

The yeast-derived product cerevisane was also demonstrated to provide protection to grape against downy mildew [32], although the active ingredient is not known. The mode of action of cerevisane was determined by transcriptomic analysis (RNA-Seq). Enzymes involved in hormone metabolism and related plant responses, defense compounds, secondary metabolites, and photosynthetic processes were up-regulated [33]. Cerevisane induced more plant defense genes than the lysate of *W. magna* C2c Maky, but had no direct activity against *P. viticola,* contrary to the lysate of *W. magna* C2c Maky. In our study, the up-regulated genes were mainly those coding the PR proteins involved in the production of defense compounds, but also the *far* gene coding the (E, E)-alpha-farnesene synthase, involved in isoprenoid synthesis, having a role in plant defense, and the glutathione S-transferase, involved in the response to oxidative stress. PR proteins are proteins that accumulate in plant tissues in response to abiotic and/or biotic stress or after a plant defense inducer treatment. PR proteins accumulate at a local and systemic level, and their expression demonstrates that plant defense mechanisms have been implemented. They have been classified into 17 families, each one having its own function, more or less known. Most of them have antimicrobial properties through hydrolytic activity on the pathogen cell wall and/or direct toxicity on the pathogen [34]. Under these experimental conditions, *W. magna* C2c Maky lysate at a dose of 3 g/L showed a moderate induction capacity of all of the tested PR protein genes at the sampling date D3. At a dose of 1 g/L, *W. magna* C2c Maky lysate induced less PR protein genes (PR1, PR4, PR5, and PR8) and only at the sampling date D2, suggesting that this concentration is too low to efficiently induce plant defenses. In the same way, a dose response and an impact of the day of treatment were also demonstrated in plant defense induction by cerevisane [33]. The best induction with our AS was obtained with the PR1 protein gene. PR1 proteins belong to the most important group of PR proteins induced by a biotic or abiotic stress. They have shown antifungal activity, especially against oomycetes such as *Phytophthora infestans* on tomato plants [35], and *Peronospora* species. Their mode of action is related to their sterol-binding activity [36] and suppression of cell death-dependent disease symptoms [37]. PR1 proteins also increase drought tolerance [38], and cerevisane was also shown to induce PR1 proteins [33], as well as COS-OGA (the internal check inducer of this study).

PR2 proteins have a glucanase action (β-1,3- glucanase) and degrade glucan [39], the main compound of bacteria and fungi cell walls. PR2 genes are induced by the salicylic acid pathway [40]. *W. magna* C2c Maky lysate only induced the synthesis of PR2 proteins at day 3 with the highest concentration tested, whereas COS-OGA produced 3-times more genes encoding PR3 proteins at day 2. Cerevisane also up-regulated these genes [33].

PR4 proteins do not have a well-known characterized function, but have shown ribonuclease and chitinase activity. Studies on wheat have suggested an antifungal activity against *Fusarium culmorum* [41,42], while other studies have reported antifungal activity on corn [43]. Genes coding PR4 proteins were up-regulated in this study by the AS and by COS-OGA, as well as by treatment with cerevisane [33], suggesting that the genes coding PR4 proteins are very sensitive to all types of foreign compounds.

PR5 proteins, also called thaumatin-like proteins (TLPs), are known to have antifungal and anti-oomycete activity, although the mechanisms by which TLPs exert these activities remain unclear. Several antifungal modes of action have been described, such as membrane permeabilization, β-glucan degradation or inhibition of enzymes such as xylanases [44]. Their genes were up-regulated by Cerevisane [33], COS-OGA, and *W. magna* C2c Maky lysate.

PR14 proteins are expressed in young leaves and are involved in lipid transfer. They seem to play a role in the transport of cutin monomers: they contribute to cutin and wax assembly [34]. They have antimicrobial properties, for example, against *Pseudomonas*, *Fusarium*, *Pythium,* and *Botrytis* [45]. Genes encoding PR14 proteins were induced by COS-OGA and our AS, as well as by cerevisane [33].

Finally, PR15 proteins have oxalate–oxidase activity with known antifungal activity, especially through the plasma membrane degradation of pathogenic fungi, thanks to a wide panel of enzymes [46]. 

In addition to these plant inducer effects, *W. magna* C2c Maky lysate demonstrated direct activity on *P. viticola,* with inhibition of both sporocyst release and zoospore germination. This direct effect was not observed with cerevisane [33] or *T. harzianum* [47]. A plant extract was shown to have both activities by activating plant defense responses and by inhibiting the release and motility of *P. viticola* zoospores [48]. This plant extract needed to be used at 5 g/L to reduce *P. viticola* sporulation under greenhouse conditions by 70%; however, the efficacy of the product was not demonstrated in the field tests. *W. magna* C2c Maky lysate was able to totally inhibit *P. viticola* sporulation in vitro at 0.3 g/L (Figure 2a). At 5 g/L, on plants in a greenhouse, it was able to decrease the severity of mildew attack by 60% (Figure 3a) when 75% of the leaves were attacked. The efficacy of the raw substance was also proven in the field tests, where an efficacy of 55% on the leaves and 41% on the bunches was observed (Figure 4a), similar to the Bordeaux mixture efficiency at 68% and 47%, respectively. Fields treated with natural substances showed reduced efficacy compared to copper with this level of mildew attack [49], confirming the potential to use the lysate of *W. magna* C2c Maky against *P. viticola*. Field trials conducted in 1995, 1997, and 1998 in Israel with β-aminobutyric acid (BABA), a natural compound, managed to effectively control grape downy mildew (>90%) when associated with a reduced dose of chemical fungicides [50]. This compound is present in numerous plants and is a pure elicitor, with no direct effect on fungi [51]. Its prohibitive price (about 7200 €/kg; Sigma-Aldrich) could explain why BABA is not authorized in the European Union. However, using raw substances in fields is not a common practice; formulated products are generally used to face environmental conditions detrimental to product efficacy, such as rain events, or to have a better handling and application of products on leaf surfaces [52]. For that purpose, we developed several formulations that were firstly tested and validated in a greenhouse (Figure 3b). Two of them were further assessed in field trials (Figure 4). The AXP01 product was tested in three field trials. In Hungary, its efficacy was similar to the AS at the same concentration (Figure 4a,b), but it provided an increased efficacy in the German trial, with 71% and 77% efficacy on the leaves and bunches, respectively (Figure 4c). It was compared to the second formulation, AXP07, in the trial conducted in France. No difference was observed on the leaves, as none of the products were efficient in this case, even the biocontrol reference Roméo^®^ (Figure 4d). AXP07 had a tendency to be more efficient than AXP01 (although not significantly) on the bunches, with 53% efficacy, whereas AXP01 at the same dose demonstrated 40% efficacy (Figure 4e). These two formulations will be improved in the coming years. To improve the use of the product and avoid the formation of a dust cloud during the solubilization process, a soluble concentrate and an oil dispersible form are under study.

To conclude, for the first time, a biocontrol product to fight downy mildew of grapevine was developed from a free-living amoeba. *W. magna* C2c Maky lysate has the rare property to possess a dual mode of action—it is a plant elicitor (indirect effect) and it inhibits the zoospore release and spore germination of *P. viticola* (direct effect). This dual mechanism is a real asset, a guarantee of reliability. Moreover, it is a safe product for human life and the environment, while some natural anti downy mildew fungicides already on the market (like sweet orange essential oil for instance) do have some toxicity and ecotoxicity weaknesses. Finally, the efficacy of the lysate of amoeba was proven to be significantly different from the untreated control in vitro, in planta, and most importantly in fields. Further studies will be conducted to determine the other target pathogens of this natural and safe product, and to position it at its best—in a practical treatment program against grapevines downy mildew.

## 4. Materials and Methods 

### 4.1. Active Substance and Formulation Preparation

*Willaertia magna* C2c Maky (ATCC PTA-7824) was axenically grown at 40 °C in a 500 L bioreactor (GE Healthcare, La Rochelle, France) filled with a Chang medium [53] without fetal calf serum. The cells were washed with NaCl 0.45%, then lysed by high-pressure homogenization at 20,000 psi (Microfluidics™, Westwood, MA, USA) and further dehydrated by lyophilization (Eurolyo, Chartres, France). The resulting powder was the raw AS. The plant protection product containing 100% AS was named AXP10. Two wettable powders (WPs) containing 50% AS were prepared from AXP10 by SBM Formulation (Bézier, France) and named AXP01 and AXP02. Two other formulated WPs were prepared from the liquid lysate and air-dried by CREATHES (Héricourt, France) to provide AXP06 and AXP07, containing 62.5% AS.

### 4.2. Study of the Stimulation of Plant Defense Genes by qPFD^®^

#### 4.2.1. Biological Material

Grapevine plants were obtained from the seeds of the Cabernet Sauvignon grape variety by Vegepolys Valley Centre R&D (Angers, France). They were maintained for 5 weeks in a growth chamber (25 °C, 16 h photoperiod), then selected at the 4–6 leaf stage and transferred to a growth chamber dedicated to the experimentation. Four modalities were tested: the *W. magna* C2c Maky lysate was tested at 1 and 3 g/L and compared to a positive control, COS-OGA [19] at 8.54 mL/L, a negative control, and the untreated control with water.

#### 4.2.2. Plant Treatment and Samplings

Experiments were performed by Vegepolys Valley Centre R&D under controlled conditions (21 °C day/18 °C night, 16 h photoperiod, 70% humidity) on grapevine plants (4–6 leaf stage). The experimental design was made of blocks of 8 plants for each modality (i.e., amoeba lysate, internal check inducer, and water control). Each modality was sprayed at day 0 (D0). At day 1 (D1), hydrogen peroxide (H_2_O_2_) was applied to each modality to mimic a pathogen attack. All treatments were carried out on both leaf surfaces, before run-off, with a compressed air sprayer.

For each modality, 8 foliar discs (6 mm diameter) were sampled by pooling 4 expanded leaves from the leaf in position three (L3) from 4 different plants at:

- D0: Initial sampling before any treatment;
- D2 and D3: Samplings performed 2 and 3 days after treatment.

Samples were deep-frozen in liquid nitrogen, and then stored at −80 °C until use. For each treatment, a backup sampling was performed and stored at −80 °C. Two independent biological repetitions were performed for the whole experiment: from sowing, treatments, up to real-time polymerase chain reaction (RT-PCR) analyses.

#### 4.2.3. Marker Genes

Gene expression analysis in the grapevine plantlets was performed by Vegepolys Valley Centre R&D using the quantitative RT-PCR microplate/DNA chip low density (qPFD^®^) method [18,54]. Briefly, after RNA extraction of the leaf samples, the yield and quality of the extracted RNA were assessed with a spectrophotometer (Nanodrop ND-1000). RNAs were then retro-transcribed into cDNA and the expression levels of 28 defense genes (Table 1) were quantified in triplicate by RT-PCR (SYBR Green) with the qPFD^®^ tool [18]. The gene expression levels were calculated using the 2−ΔΔCt method, i.e., relative to a calibrator (i.e., the untreated D0 sample), and normalized by the geometric mean of the relative expressions of 3 reference genes (i.e., *tuA*, actin, and GAPDH) [18]. A log2 transformation of the data was applied in order to provide the same weight to the induction and repression of the genes. The water control plants sampled the same day were analyzed and used as a reference to determine the relative expression.

### 4.3. Anti-Oomycetal Properties of the AS 

These assays were performed by the STAPHYT-L&G team (Laboratory and Glasshouse, Martillac, France).

#### 4.3.1. Oomycete Strain

The oomycete material consisted of a mono-sporocyst strain of downy mildew, from an internal collection created and maintained by STAPHYT-L&G. It has a normal sensitivity to the main families of fungicides used to control grape downy mildew. As part of this program, preliminary steps were performed to replicate and multiply this strain in order to produce sufficient oomycete material for testing.

#### 4.3.2. Active Substance Preparation

A stock solution at 10 g/L was prepared, and serial dilutions were carried out with demineralized water by using an automatic dilutor (Miniprep 60, Tecan, Männedorf, Switzerland). Eight concentrations were studied: 0.03, 0.05, 0.1, 0.3, 0.5, 1, 3, and 5 g/L. The control condition was performed with demineralized water. Each condition was repeated five times.

#### 4.3.3. In Vitro Test

The microplates were inoculated with the spore suspension mixed with the different studied doses. Fresh sporangia were harvested from contaminated leaves with a paint brush and suspended in demineralized water. The concentration of the inoculum was adjusted to 10^5^ non-granulated sporangia per milliliter. This sporocyst suspension was thereafter half-diluted by mixing it with an equal volume of two-fold concentrated AS solutions. Once inoculated, the microplates were incubated in an air-conditioned chamber for 6 h. After this time, the development of the spores was blocked by an amendment of a 10 μL drop of cotton blue. The microplates were then stored in a cool place until they were rated. An evaluation of the percentage of the sporocyst release and the zoospore germination percentage was performed on each of the 5 wells per AS concentration. The assessment was made on 100 sporocysts or zoospores per well. Each well received a percentage score, and an average of the 5 wells was then calculated, giving the percentage of release and the percentage of germination.

#### 4.3.4. In Vivo Test

The plant variety used for the tests was that of Cabernet Sauvignon. The plants from which the leaves were taken were produced in a greenhouse under conditions that ensured that the quality of the plants and their health status were controlled throughout the year. Leaf discs with a diameter of 18 mm were cut using a punch. A two-step process was used to study the efficacy of the AS on leaf discs.

- Evaluation of the disease development on leaf discs

Ten discs were placed into 90 mm diameter Petri dishes (per condition). Three 10 μL droplets of the AS and the sporocyst mixture were deposited on the abaxial (lower) surface of each leaf disc. After inoculation, the Petri dishes were placed into a climatic chamber (21 ± 2 °C, 14/10 h light/darkness period) for 8 days. The moisture necessary for the development of infection was supplied inside the dishes by a wet filter paper. The downy mildew damage on the leaf discs was assessed after the incubation period.

- Evaluation of the contamination properties of spores produced on leaf discs

After the assessment, the spores on each of the contaminated leaf discs were suspended in demineralized water. This suspension was quantified and then used to inoculate new Cabernet Sauvignon leaf discs to evaluate the contaminating potential of the spores that were produced on leaves treated with the AS. A control was inoculated with the spores produced on the untreated leaf discs. Once contaminated, the Petri dishes containing the leaf discs were placed into a climatic chamber (21 ± 2 °C, 14/10 h light/darkness period) for 8 days. At the end of this incubation period, the downy mildew damage assessment on the leaf discs was recorded. The results of the sporulation scores were analyzed to calculate the efficacy for each concentration studied. An average of the disease severity was calculated from the results obtained from the 10 discs.

Each drop impact deposited on the leaf discs was scored with an average damage score, ranging from 0 to 4, taking into account the surface colonized by the oomycete and the intensity of sporulation with 0 corresponding to the absence of mildew development, 1 to low contamination, 2 to moderate contamination, 3 to high contamination, and 4 to full surface contamination. On each leaf disc, 3 droplets were deposited; thus, 3 scores per leaf were obtained. Finally, for a given concentration, 30 scores were obtained to characterize the efficacy. The efficacy of each rate was obtained by applying the following formula:

$$E=\frac{\left(T-D\right)}{T}\times 100$$

where *T* is the average disease severity on the 10 control discs, and *D* is that observed on the discs treated with the fungicide at different doses.

### 4.4. Greenhouse Tests

#### 4.4.1. Plant Material

The trials were carried out on young Cabernet Sauvignon plants produced in a greenhouse (STAPHYT-L&G) from one-eyed shoots. These plants were grown in a greenhouse under conditions that ensured the control of the quality of the plants and their health status during their cultivation. Homogeneous batches of 6 plants were formed for the study of each modality of the test. Each plant was staked, just before the application of the treatment, and the leaf stages were identified using a colored marker placed under the third leaf spread. This later allowed, during the assessment, an identification of the treated and untreated leaves. At the end of the rating, an analysis of the results was carried out on the 5 plants constituting the most homogeneous batch (the least significant variation around the average of the symptoms).

#### 4.4.2. AS Application

According to the experimental design, the application mode comprised the spraying of the AS on the whole plant. Both the upper and lower surfaces of the leaves were treated. The spraying was performed in a preventive manner, 24 h before infection. A classical application method by spraying was used: The plants were placed in a spray booth equipped with a spray bench carrying 5 nozzles (2 lateral right, 2 lateral left, and 1 upper). This bench moved on a guidance rail at a constant speed. The pressure at which spraying was applied and the speed of the bench were a function of the volume to be supplied. In this study, the volume of water used was 600 L/ha. The different solutions tested were AXP10 at 1, 3, and 5 g/L compared to the Bordeaux mixture (RSR Disperss) at 6.25 g/L in the first trial. A second trial was performed to compare the AS AXP10 with several formulations with 3 g/L of AS. AXP01 and AXP02 contained 50% AS and were thus sprayed at 6 g/L, while AXP06 and AXP07 contained 62.5% AS and were thus sprayed at 4.8 g/L. AXP10 at 3 g/L was also tested in combination with HELIOSOL^®^ (ActionPin, Castets, France) at 1.25 mL/L.

#### 4.4.3. *Plasmopara viticola* Inoculation

The fungal material consisted of a mono-sporocyst strain of downy mildew from an internal collection created and maintained by STAPHYT-L&G. It has normal sensitivity to the main families of fungicides used to control grape downy mildew. A suspension of sporocysts was prepared by washing the infected leaves with demineralized water at a low temperature in order to block the release of zoospores. The spores of the fungus were thus harvested and then titrated with a Malassez cell. The optimal concentration was 25,000 spores/mL. The plants were inoculated by spraying a sporangial suspension on the lower surface of the leaves. Each plant was treated separately and was contaminated on each leaf stage.

#### 4.4.4. Incubation and Notations

After their contamination, the 6 plants of the same condition were gathered inside a hermetic enclosure in order to maintain a saturated relative humidity, necessary for the development of the disease. This device also made it possible to isolate the different test conditions from one another. The isolated plant batches were placed in an air-conditioned room (21 ± 2 °C, 14 h of light). A few hours after inoculation (~8 h), the incubation chambers were removed. The plants remained in incubation for 7 days under the same light and temperature conditions as before. At the end of this period, the incubation chambers were replaced for each condition to allow the expression of the pathogen’s sporulation, and 24 h after the replacement of the enclosures, an estimate of the severity of the disease was established for each plant and for each leaf stage (from L4 to L1 + N, N being the new non treated leaves that appeared during the 7 days of incubation).

The assessment was based on visual observations of the fungus damage, by considering two parameters: the foliar surface colonized by the fungus and the intensity of sporulation. We used a scale from 0% to 100% (with an increase of 5% by 5%). Each leaf was observed individually. For each plant, the disease severity was calculated, only taking into account the score assigned for the leaves L2, L3, and L4. The leaves were numbered before the treatment with L1 which was the most recent leaf at the top of the plant. Then, for each condition of the test, a mean disease severity score was calculated by averaging the attack rates obtained for the 5 plants of the same condition (elimination of the plant with the disease severity furthest from the average disease severity). From this mean disease severity score, the efficacy (*E*) of the product for each condition could be calculated by comparison to the mean disease severity score obtained in the control condition (untreated plants) according to the formula:

$$E=\frac{\left(T-D\right)}{T}\times 100$$

where *T* is the mean disease severity score obtained for the control condition (untreated plants), and *D* is that observed on the plants treated with the fungicide at different doses.

### 4.5. Field Tests

Three trials were conducted in 2019 to assess the efficacy against downy mildew on grapes of the raw AS, AXP10, and the two formulated products, AXP01 (containing 50% AS) and AXP07 (containing 62.5% AS). One efficacy trial was conducted in Hungary, one in Germany, and one in France. 

The observations were focused on the incidence and severity of the disease on grapevine bunches and leaves. The trial design, test compound efficacy evaluations, and phytotoxicity assessments were all performed in compliance with the principles of good experimental practices according to the European and Mediterranean Plant Protection Organization (EPPO) guidelines, which define the standard procedures for the evaluation of plant protection products. The application and evaluation dates were expressed according to the Biologische Bundesanstalt, Bundessortenamt and CHemical industry (BBCH) code [55], corresponding to specific developmental stages of the plant. For example, BBCH 77 corresponds to bunch closure and BBCH 81 to the beginning of berry ripening.

#### 4.5.1. Field Test Conducted in Hungary

SynTech Research Hungary Ltd. (Szombathely, Hungary) investigated the efficacy of AXP10 at 0.5, 1, and 2 kg/ha in comparison to AXP01 at 2 kg/ha and to copper (BORDÓMIX DG at 3.75 kg/ha) against downy mildew of grapevine (*Plasmopara viticola*) near Gyékényes in the southwest of Hungary in 2019 (46.258073, 16.96484). The grapevine variety was Chardonnay, and the age of the plantation was 10 years. The treatments were applied in four replications on small plots. The treatments were applied six times on 6th June 2019 at BBCH 57, on 12th June 2019 at BBCH 60, on 18th June 2019 at BBCH 65, on 23rd June 2019 at BBCH 69, on 28th June 2019 at BBCH 71, and on 3rd July 2019 at BBCH 71 of the crop. As application equipment, an electronic pump backpack sprayer was used with a 500 L/ha spray volume. The number of spots caused by downy mildew was counted per plot. The percentage of the severity and the incidence of the disease on the leaves and bunches were also assessed.

#### 4.5.2. Field Test Conducted in Germany

Hetterich Fieldwork GbR (Schwarzach am Main, Germany) investigated the efficacy of AXP10 at 0.5, 1, and 2 kg/ha in comparison to AXP01 at 2 kg/ha and to copper oxychloride (Cuprozin^®^ Progress at 0.4 to 1.6 L/ha) against *P. viticola* under natural conditions in a German vineyard located in Sulzfeld in Bavaria (49.70766, 10.116076). The grapevine variety was Müller Turgau. The first application took place on 7th of June at BBCH 57 of the crop. The next applications were performed on 14th June at BBCH 65, 21st June at BBCH 69, 27th June at BBCH 73, 5th July at BBCH 75, 10th July at BBCH 77, 18th July at BBCH 79, and 26th July at BBCH 81. This trial was conducted with an artificial infestation performed on 13th June. The plants were sprayed with a sporal solution. The sprayed leaves were then packed in plastic bags for 12 h. After the 12 h, the bags were removed. The infection worked very well, and the first infestation on the leaves was visible on the 24th of June. The number of spots caused by downy mildew was counted per plot. The percentage of the severity and the incidence of the disease on the leaves and bunches were also assessed.

#### 4.5.3. Field Test Conducted in France

Sciences Agro Atlantique (Saint-Germain-du-Puch, France) investigated the efficacy of AXP01 at 1, 2, 4, and 6 kg/ha in comparison to AXP07 at 3.2 kg/ha, to copper (BM RSR Disperss^®^ at 3.75 g/ha), and to a biological control product (Roméo^®^ at 0.25 kg/ha) against *P. viticola* under natural conditions in a French vineyard located in Moulon in the southwest of France (44.860109, −0.22577). The grapevine variety was Merlot. The fungicides were applied every 6 to 8 days from 29th April, at the crop stage of the fifth unfolded leaf, until 9th July at bunch closure, for a total of 11 applications. An artificial infection was produced on a buffer plant on 9th May, 3 days after the second application. Three leaves on the plant buffer stock (not included in the study) between plots were infested with a solution of 60,000 spores/mL of a local environmental *P. viticola* strain. The number of spots caused by downy mildew was counted per plot. The percentage of the severity and the incidence of the disease on the leaves and bunches were also assessed.

### 4.6. Statistical Analyses

Statistical significance was determined through the use of analysis of variance (ANOVA) (Kruskal–Wallis test and multiple pair-wise comparison Dunn test).

## Figures and Tables

**Table 1:** List of the plant defense genes studied [18].

Defence Classes and Subclasses		Defence Genes	
		Gene Codes	Complete Names
Chemical and/or physical barriers	PR proteins	PR-1	Pathogenesis-related protein 1
		PR-2	Pathogenesis-related protein 2 (glucanases)
		PR-4	Pathogenesis-related protein 4 (hevein-like)
		PR-5	Pathogenesis-related protein 5 (thaumatin-like, osmotin)
		PR-8	Pathogenesis-related protein 8 (class III chitinase)
		PR-14	Pathogenesis-related protein 14 (lipid transfer protein)
		PR-15	Pathogenesis-related protein 15 (oxalate oxidase)
	Phenylpropanoids	PAL	Phenylalanine ammonia-lyase
		CHS	Chalcone synthase
		DFR	Dihydroflavonol reductase
		ANS	Anthocyanidin synthase
		PPO	Polyphenol oxidase
	Isoprenoids	HMGR	Hydroxymethyl glutarate-CoA reductase
		FPPS	Farnesyl pyrophosphate synthase
		Far	(E,E)-alpha-farnesene synthase
	Cysteines	CSL	Alliinase
	Oxidative stress	APOX	Ascorbate peroxidase
		GST	Glutathion S-transférase
		POX	Peroxidase
	Parietal modification	CalS	Callose synthase
		Pect	Pectin methyl esterase
		CAD	Cinnamyl alcohol dehydrogenase
Hormonal signaling	Salicylic acid (SA)	EDS1	Disease resistance protein EDS 1
		WRKY	WRKY transcription factor 30
	Jasmonic acid (JA)	LOX2	Lipoxygenase AtLOX2
		JAR	Jasmonate resistant 1
	Ethylene (ET)	ACCO	1-aminocyclopropene-1-carboxylate oxidase
		EIN3	EIN3-BINDING F BOX PROTEIN 1

## Supplementary Materials

The following are available online at https://www.mdpi.com/2223-7747/9/8/1013/s1: Table S1: Toxicological studies performed to assess the hazard of the active substance “Lysate of *Willaertia magna* C2c Maky” to human health; Table S2: Ecotoxicological studies performed to assess the hazard of the active substance “Lysate of *Willaertia magna* C2c Maky” to the environment.
