# Supplementary material for: A New Active Substance Derived from Lyzed Willaertia magna C2c Maky Cells to Fight Grapevine Downy Mildew
Source: Plants (Basel). 2020 Aug 11;9(8):1013. doi: 10.3390/plants9081013 (PMC7463879; doi:10.3390/plants9081013)
Supplement: Supplementary file 1 [file plants-09-01013-s001.pdf]

Table S1. Toxicological studies performed to assess the hazard of the active substance 'Lysate of *Willaertia magna* C2c Maky' to human health. The studies have been performed in 2019 and 2020 by independent laboratories under Good Laboratory Practices. Test item was the pure 'Lysate of *Willaertia magna* C2c Maky'.

**Toxicological Study**	**Endpoint** [1]
Acute oral toxicity (OECD 423)	LD_{50} oral – rat > 2000 mg/kg b.w.
Acute oral toxicity (OECD 408)	NOAEL oral rat – 28 d = 1000 mg/kg b.w.
Acute inhalation toxicity (OECD 403)	LC_{50} inhalation – 4 h > 0.773 mg/L
Skin corrosion/irritation (OCED 439)	Non-irritant.
Serious eye damage/irritation (OCED 438)	Non-irritant.
Respiratory or skin sensitization (OECD 442B)	Not skin-sensitizing.
Germ cell mutagenicity (OECD 471 and OECD 487)	Non mutagenic. Neither clastogenic nor aneugenic.

[1] LD_{50}: lethal dose 50%, the amount of a test substance that is sufficient to kill 50 percent of a population of animals within a certain time. NOAEL: no observed adverse effect level, the highest experimental point tested that is without adverse effect. LC_{50}: lethal concentration 50%, the average concentration of a test substance capable of killing 50% of the test animals exposed by inhalation under specific conditions.

Table S2. Ecotoxicological studies performed to assess the hazard of the active substance 'Lysate of *Willaertia magna* C2c Maky' to the environment. The studies have been performed in 2019 and 2020 by independent laboratories under Good Laboratory Practices. Test item was the pure 'Lysate of *Willaertia magna* C2c Maky'.

Toxicological Study	Endpoint [1]
Acute toxicity to fish (*Oncorhynchus mykiss,* OECD 203)	EC_{50} – 96h > 100 mg/L NOEC = 100 mg / L
Acute toxicity to aquatic invertebrates (*Daphnia magna,* OECD 202)	EC_{50} – 48h > 100 mg/L NOEC – 48h = 100 mg/L
Chronic toxicity to aquatic invertebrates (*Daphnia magna,* OECD 211)	21-d NOAEC = 10.0 mg/L 21-day EC_{10} > 10.0 mg/L
Acute toxicity to algae (*Pseudokirchneriella subcapitata,* OECD 201)	72-h EC_{50} > 100 mg/L 72-h NOEC (growth rate and yield) = 100 mg/L
Acute contact toxicity to bees (OECD 214; OCSPP 850.3020)	NOEL = 100 µg/bee<br>LD_{50} > 100 µg/bee
Acute oral toxicity to bees (OECD 213)	NOEL = 100 µg /bee<br>LD_{50} > 100 µg/bee
Oral chronic toxicity to bees (OECD 245)	10d-NOEC > 277.8 mg/kg of feeding solution<br>NOEDD – 10 d = 10.31 µg/bee/day<br>10d-LDD_{50} > 10.31 µg/bee/day
Toxicity to earthworm – sub-lethal effects (OECD 222)	LC_{50}-28d > 1800 mg/kg (dry mass of soil)<br>NOEC reproduction after 56 days = 1000 mg/kg (dry mass of soil)<br>EC_{10} reproduction = 1631 (954–1676) mg/kg (dry mass of soil)
Toxicity to birds (*Coturnix coturnix japonica,* OECD 223; OCSPP 850.2100)	LD_{50} oral - 14 d > 2000 mg/kg b.w.<br>NOEL oral - 14 d = 2000 mg/kg b.w.
Persistence and degradability (OECD 301)	Ready biodegradable

[1] Legend: EC_{50} / EC_{10}: concentration of test substance which results in a 50 percent / resp. 10 percent reduction in either algae growth or Daphnia immobilization. NOEC/NOEL: no observed effect concentration / level, concentration/level of a test substance in an environmental compartment (water, soil, etc) which below an unacceptable effect is unlikely to be observed. LD_{50}: lethal dose 50%, the amount of a test substance that is sufficient to kill 50 percent of a population of animals within a certain time. NOEDD: No observed effect dietary dose. LDD_{50}: median lethal dietary dose.
